# Dithymoquinone Analogues as Potential Candidate(s) for Neurological Manifestation Associated with COVID-19: A Therapeutic Strategy for Neuro-COVID

**DOI:** 10.3390/life12071076

**Published:** 2022-07-19

**Authors:** Afrasim Moin, Bader Huwaimel, Ahmed Alobaida, Mohammed Khaled Bin Break, Danish Iqbal, Rahamat Unissa, Qazi Mohammad Sajid Jamal, Talib Hussain, Dinesh C. Sharma, Syed Mohd Danish Rizvi

**Affiliations:** 1 Department of Pharmaceutics, College of Pharmacy, University of Hail, Hail 81442, Saudi Arabia; a.moinuddin@uoh.edu.sa (A.M.); a.alobaida@uoh.edu.sa (A.A.); srunissa@gmail.com (R.U.); 2 Department of Pharmaceutical Chemistry, College of Pharmacy, University of Hail, Hail 81442, Saudi Arabia; b.huwaimel@uoh.edu.sa (B.H.); m.binbreak@uoh.edu.sa (M.K.B.B.); 3 Department of Medical Laboratory Sciences, College of Applied Medical Sciences, Majmaah University, Majmaah 11952, Saudi Arabia; da.mohammed@mu.edu.sa; 4 Department of Health Informatics, College of Public Health and Health Informatics, Qassim University, Al Bukayriyah 52741, Saudi Arabia; m.quazi@qu.edu.sa; 5 Department of Pharmacology and Toxicology, College of Pharmacy, University of Hail, Hail 81442, Saudi Arabia; 6 School of Life Sciences, The Glocal University, Saharanpur 247121, Uttar Pradesh, India; ddcsharma@gmail.com; 7 Department of Microbiology, School of Life Sciences, Starex University, Gurugram 122413, Haryana, India

**Keywords:** COVID-19, neuro-COVID, SARS-CoV-2, dithymoquinone, molecular dynamics

## Abstract

The COVID-19 era has prompted several researchers to search for a linkage between COVID-19 and its associated neurological manifestation. Toll-like receptor 4 (TLR-4) acts as one such connecting link. spike protein of SARS-CoV-2 can bind either to ACE-2 receptors or to TLR-4 receptors, leading to aggregation of α-synuclein and neurodegeneration via the activation of various cascades in neurons. Recently, dithymoquinone has been reported as a potent multi-targeting candidate against SARS-CoV-2. Thus, in the present study, dithymoquinone and its six analogues were explored to target 3CL^pro^ (main protease of SARS-CoV-2), TLR4 and PREP (Prolyl Oligopeptidases) by using the molecular docking and dynamics approach. Dithymoquinone (DTQ) analogues were designed in order to investigate the effect of different chemical groups on its bioactivity. It is noteworthy to mention that attention was given to the feasibility of synthesizing these analogues by a simple photo-dimerisation reaction. The DTQ analogue containing the 4-fluoroaniline moiety [Compound (**4**)] was selected for further analysis by molecular dynamics after screening via docking-interaction analyses. A YASARA structure tool built on the AMBER14 force field was used to analyze the 100 ns trajectory by taking 400 snapshots after every 250 ps. Moreover, RMSD, RoG, potential energy plots were successfully obtained for each interaction. Molecular docking results indicated strong interaction of compound (**4**) with 3CL^pro^, TLR4 and PREP with a binding energy of −8.5 kcal/mol, −10.8 kcal/mol and −9.5 kcal/mol, respectively, which is better than other DTQ-analogues and control compounds. In addition, compound (**4**) did not violate Lipinski’s rule and showed no toxicity. Moreover, molecular dynamic analyses revealed that the complex of compound (**4**) with target proteins was stable during the 100 ns trajectory. Overall, the results predicted that compound (**4**) could be developed into a potent anti-COVID agent with the ability to mitigate neurological manifestations associated with COVID-19.

## 1. Introduction

COVID-19 was initially reported in the city of Wuhan, China, two years ago, and subsequently the search for anti-COVID drugs started. Tracking of COVID-19 clinical manifestations suggests that not only respiratory system failure but also its strong association with other organs such as the brain, the heart, the gut, the liver and the kidney leads to the patients’ grave condition [1]. However, neurological involvement during and after COVID-19 is one of the most common manifestations of the infected patients [2,3]. The scientific community is tracing the underlying mechanism of correlation between COVID-19 and its linked neurological symptoms. Toll-like receptor 4 (TLR-4) appears to be one such connecting link, as the spike protein of SARS-CoV-2 (the COVID-19 causative agent) could bind with equal efficacy with ACE-2 (angiotensin-converting enzyme 2) and TLR-4 receptors of dopaminergic neurons [4,5]. In fact, the cytokine storm generated after TLR-4 activation/binding with the spike protein of SARS-CoV-2 causes neuro-inflammation and neuro-degeneration in COVID-19 patients [4,5,6,7]. Thus, targeting TLR-4 may be effective [4,8,9]. It is to be noted that our team recently discovered dithymoquinone (DTQ) as a potent dual-targeting agent against SARS-CoV-2 [10]. Furthermore, different DTQ analogues were designed to explore their potency against 3CL^pro^ (3-chymotrypsin like protease), TLR4 and PREP (prolyl oligopeptidases) in the current study.

3CL^pro^ is the main protease enzyme of SARS-CoV-2 that plays a crucial role in its replication [11,12]. In fact, 3CL^pro^ cuts coronavirus polypeptides in 11 different positions that create several non-structural proteins pertinent to its replication [13]. Hence, 3CL^pro^ is considered as a potential target for designing new COVID-inhibitors [11,13,14]. Increased expression of ACE2 in oligodendrocytes, neurons and astrocytes could aid in the neuro-invasion of SARS-CoV-2 [15,16]. In addition, SARS-CoV-2’s ability to activate TLR-4 could predispose COVID-19-infected patients to neuro-inflammation, neurodegeneration and alpha-synuclein aggregation [4,5]. However, alpha-synuclein aggregation could be reduced by inhibiting PREP enzyme activity [17]. Thus, exploring new SARS-CoV-2 active drug candidates against TLR-4 and PREP may help to mitigate neurological manifestations associated with COVID-19.

In the present study, DTQ analogues [compound (**1**) to compound (**7**)] were designed in order to investigate the effect of different chemical groups (bromine, chlorine, methylamine, 4-fluoroaniline, methylbromo and methylchloro) on their bioactivity [18]. It is also crucial to note that care was given to the feasibility of synthesizing these designed analogues, as all of them may be synthesized in a simple photo-dimerization reaction. All the seven analogues of DTQ and DTQ (without any modification) were subjected to molecular docking, and physicochemical and toxicity analysis. Further, the dynamic simulation analysis was applied for the screened-out potential DTQ analogue to study the stability during the 100 ns trajectory.

## 2. Materials and Methods

### 2.1. Designing DTQ Analogues

**Figure 1:**
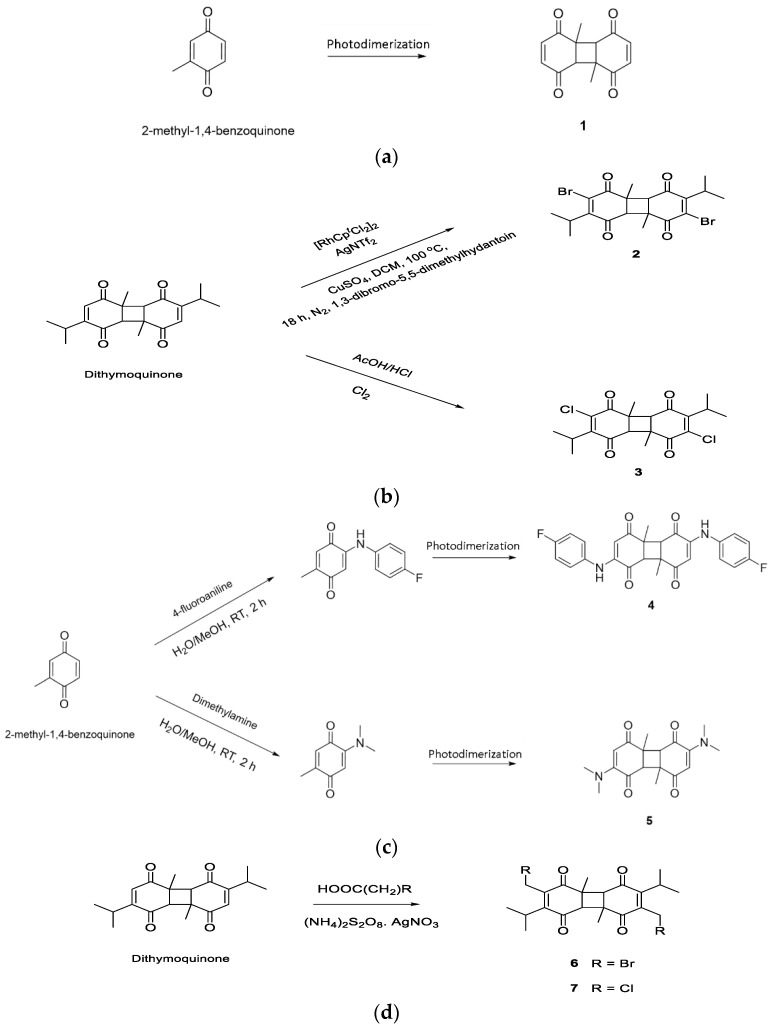
Schematic representation of the designing of different DTQ analogues. (**a**) DTQ analogue without isopropyl groups, (**b**) Dibrominated and Dichlorinated DTQ analogues, (**c**) DTQ analogues with the addition of 4-fluoroaniline and dimethylamine, (**d**) DTQ analogues with the addition of methylbromo and methylchloro.

Photodimerization is used to successfully synthesize DTQ from thymoquinone [19]. Thus, commercially available 2-methyl-1,4-benzoquinone undergoes photodimerization under fluorescent light to synthesize compound (**1**) that is a DTQ analogue without isopropyl groups (Figure 1a). Dibrominated DTQ analogue [compound (**2**)] is synthesized via direct bromination of dithymoquinone (Figure 1b), and this is based upon a previous study which showed that 1,4-benzoquinones may be directly brominated under the presence of a rhodium-based catalyst [20]. Similarly, dichlorinated DTQ analogue [compound (**3**)] was directly synthesized by using chlorine in acidic medium (Figure 1b). Compound (**4**) was DTQ analogue with the addition of 4-fluoroaniline to each of the quinone rings and compound (**5**) was DTQ analogue with the addition of dimethylamine to each of the quinone rings. Both compounds (**4**) and (**5**) were synthesized by using the commercially available 2-methyl-1,4-benzoquinone as a starting material, as reported previously [18]. The resulting compounds were then subjected to photodimerization (Figure 1c). Compound (**6**) was DTQ analogue with the addition of methylbromo to each of the quinone rings and compound (**7**) was DTQ analogue with the addition of methylchloro to each of the quinone rings. Compounds (**6**) and (**7**) were synthesized by direct haloalkylation of the dithymoquinone (Figure 1d), based on a previously reported synthesis methodology [21]. However, DTQ without any modification was named as compound (**8**).

### 2.2. Physicochemical Parameters and Toxicity Prediction

All the compounds were subjected to physicochemical parameter analysis and toxicity prediction by the Osiris DataWarrior property explorer tool. Molecular properties were used to evaluate the Lipinski violation [22] and calculate the % oral absorption [23] of each compound. 

The following Equation (1) was used to calculate the % oral absorption based on TPSA:

(1) % Absorption=109−(0.345×TPSA) 

### 2.3. Molecular Docking and Interaction Analysis

#### 2.3.1. Target Protein Preparation

Target proteins 3CL^pro^ (PDB ID: 6LU7), TLR4 (PDB ID: 3FXI) and PREP (PDB ID: 3DDU) were obtained from the protein data bank. A PDB (gz) file for each target was downloaded from the protein data bank and visualized by aDiscovery Studio visualizer tool. Hetero atoms (including control ligands) were deleted from the target proteins. All target proteins were further saved in a PDB format; however, they were converted into a PDBQT format before being subjected to a run for docking experiments. Rizvi et al.’s [24] method was applied to convert the target proteins into a PDBQT format using the AutoDock 4.2 program. Prior to conversion into a PDBQT format, polar hydrogen, solvation parameters and Kollman united atom charges were added to the target protein structure.

#### 2.3.2. Ligand Preparation

The structure of all designed DTQ analogues [compounds (**1**) to (**7**)] was prepared by ChemDraw and saved in SDFformat. However, structures for compound (**8**) (ID: 398941), Lopinavir (ID: 92727), Resatrovid (ID: 11703255) and Berberine (ID: 2353) were retrieved from the PubChem database. The 3-D structures obtained from the PubChem database were also in SDF format. An OpenBabel tool was used to convert all the ligand structures from SDF format into a PDBQT format before subjecting them to docking via AutoDock Vina.

#### 2.3.3. Molecular Docking

Molecular docking of each compound with the target proteins was performed using the AutoDock Vina platform [25]. Grid coordinates were pre-defined for each protein to target the active sites. The grid box size was kept as 60 × 60 × 60, whereas the center of the grid box was set as x: −16.539; y: 15.246; z: 67.334 for 3CL^pro^, x: 9.261; y: 0.905; z: 20.315 for TLR4 and x: −8.263; y: 14.166; z: 27.480 for PREP, respectively. The AutoDock Vina results were displayed in terms of affinity (kcal/mol). The algorithm for each interaction was divided into 10 modes in descending order. However, Pymol software was used to save the complex of the best docking confirmation. Detailed interaction of each complex was further analyzed by the Discovery Studio Visualizer tool. 

### 2.4. Molecular Dynamics Simulation Analysis

A docked complex of compound (**4**) with 3CL^pro^, TLR-4 and PREP was used for dynamic simulation study by applying the YASARA-structure tool [26]. The boundary of the simulation cell (20 Å around the target protein) was kept periodic with the help of AMBER14 force field and solvent (water) was filled at 0.998 g/mL density. Further, the protonated state of the protein was tuned at pH 7.4 through pKa prediction, and the optimization of the H-bond network was performed to increase solute stability [27]. Moreover, NaCl ions were added to the system for environment neutralization. Energy minimization of the system was performed by a YASARA-structure tool to resolve the bumps and covalent geometry. The steepest descent and simulation annealing approaches were used to remove clatters before starting the 100 ns trajectory of simulation by ‘AMBER14 force field‘ for solute [28], ‘AM1BCC [29] and GAFF2 [30]’ for compound (**4**), and ‘TIP3P’ for water. A cut-off of 8Å was used for the van der waals force, whereas the particle mesh Ewald algorithm and electrostatic forces were used without a cut-off. Equilibration of the system was performed by position restraining via applying NPT ensembles [31]. All three complexes were equilibrated with 2.5 fs and 5.0 fs multi-time-steps for bonded and non-bonded interactions at 298 K temperature and pressure 1 bar. During the complete simulation process, NPT ensembles iso-thermal and -baric environments were maintained. A Berendsen thermostat time average -temperature and -pressure approach was used to control the temperature and pressure of the system [32]. Bond and angles constraints were maintained by the multi-step-algorithm approach via the modified version of LINCS [33]. A user-friendly interphase ‘md_runfast.mcr’ of YASARA-structure macros tool was applied to complete the simulation process, and ‘md_analyze.mcr’ was applied for trajectory analysis. A total of 400 snapshots were taken after every 250 ps to generate the Figures using the YASARA-structure tool. 

## 3. Results and Discussion

Different complications and multi-organ involvement are common in SARS-CoV-2-infected patients [1]. However, the most prominent manifestation after respiratory symptoms is a change in neurological behavior [2,3]. The Toll-like receptor 4 (TLR-4) and prolyl oligopeptidase (PREP) may act as a connecting link for the neurological symptoms associated with COVID-19 [4,5]. In a recent study, dithymoquinone (DTQ) was observed as a most potent inhibitor of SARS-CoV-2 enzymes among the different natural compounds tested [10]. In a step forward, the present study used DTQ scaffolds to design seven compounds [compound (**1**) to compound (**7**)] to explore their potential against TLR-4, PREP and 3CL^pro^. The findings help to predict the best plausible option in the form of dithymoquinone analogue [compound (**4**)] to mitigate the neurological effects of COVID-19.

### 3.1. DTQ Analogues Designing

In the present study, DTQ analogues were designed in order to investigate the effect of different chemical groups on their bioactivity to develop them into highly potent agents. Initially, the investigation was performed to observe the effect of halogenation on the biological activity of DTQ via designing the bromo [compound (**2**)] and chloro [compound (**3**)] derivatives. It has been previously reported [18] that brominated and chlorinated thymoquinone analogues resulted in bio-activity enhancement, so the case may be similar to designed halogenated DTQ analogues. In addition, we wanted to investigate the biological effect of increasing the chain length of the halogenated derivatives, and this led us to design the DTQ analogue with the addition of methylbromo [compound (**6**)] and methylchloro [compound (**7**)] to each of the quinone rings. 

It was previously shown [18] that the addition of a dimethylamine group to thymoquinone also resulted in bioactivity-enhancement against ovarian cancer cells. This prompted us to investigate the effect of adding nitrogen-containing substituents. The DTQ analogue [compound (**5**)] was designed with the addition of dimethylamine to each of the quinone rings. A further related nitrogen-containing analogue [compound (**4**)] was also designed via the incorporation of the 4-fluoroaniline group, with increased hydrophobicity and bulkiness. Comparing the activities of these two molecules predicted the importance of nitrogen-containing groups in general; in addition to the polarity and size that these groups possess to yield the optimum bioactivity.

Moreover, to investigate the importance of DTQ isopropyl groups to bioactivity, compound (**1**) without isopropyl groups was also designed. The isopropyl groups may enhance target engagement via further hydrophobic and van der Waals interactions with the target; however, there is also the possibility that it may result in steric hindrance thus preventing optimum interactions with the target receptor. Furthermore, all the designed DTQ analogues were subjected to toxicity prediction and physicochemical property assessment.

### 3.2. Physicochemical Properties and Toxicity Potential Prediction of DTQ Analogues

The physicochemical properties of all DTQ analogues, DTQ and control compounds were calculated by using the Orisis property calculator tool (Table 1). On the basis of physicochemical properties, the Lipinski violation [22] for each compound was estimated. Only lopinavir (used as a control for 3CL^pro^ enzyme of SARS-CoV-2) violated the Lipinski rule; however, all other compounds did not violate it. Our aim was to explore DTQ analogues as anti-COVID agents that could mitigate the neurological manifestation associated with COVID-19. The drugs that are targeted towards the CNS have a cut-off of 90 Å^2^ for TPSA (total polar surface area) and a molecular weight below 450 [34,35]. Thus, TPSA was also calculated for each compound. Here, almost all the compounds showed TPSA under the acceptable range except lopinavir, whereas the molecular weight range was exceeded for compound (**2**), compound (**6**) and lopinavir. Moreover, oral absorption of a drug is an important parameter that was calculated with the help of TPSA by using the approach of Zhao et al. [23]. All the compounds showed more than 70% absorption except lopinavir.

Toxicity was predicted for each compound (Table 1) using the Orisis tool to assess the mutagenicity, tumorigenicity, reproductive effect and irritability. Out of all the compounds, compound (**1**), compound (**2**), compound (**4**), compound (**5**) and compound (**8**) showed no toxicity. To select the best potent DTQ analogue, the designed analogues were further analyzed for their molecular interactions with target proteins.

### 3.3. Molecular Docking Analysis

Molecular docking of DTQ analogues was performed by the AutoDock Vina and interaction analysis was done on a Discovery studio visualizer. Table 2 shows the results of DTQ analogues interaction with 3CL^pro^, TLR-4 and PREP. Compound (**4**) showed better interaction than positive control (lopinavir) with the active site of 3CL^pro^. Gibbs free energy (ΔG) of ‘Compound (**4**)- 3CL^pro^‘ interaction was estimated as −8.5 kcal/mol. On the other hand, all the designed DTQ analogues showed better interaction with TLR-4 except compound (**5**) in comparison to positive control (resatrovid). However, among the different DTQ analogues, compound (**4**) showed strong interaction with TLR-4, where ΔG was −10.8 kcal/mol. Similarly, compound (**4**) interaction with PREP (ΔG = −9.5 kcal/mol) was better than positive control (berberine). All these results indicated that compound (**4**) was a more active inhibitor than other DTQ analogues. In other words, the addition of 4-fluoroaniline to each of the quinone rings markedly enhanced the potency of DTQ. Further, Discovery studio was used to obtain a deep insight into the interaction of compound (**4**) with the target proteins, and validation re-docking experiments were also performed.

### 3.4. Analysis of Interaction of Compound (***4***) with Target Proteins

**Figure 2:**
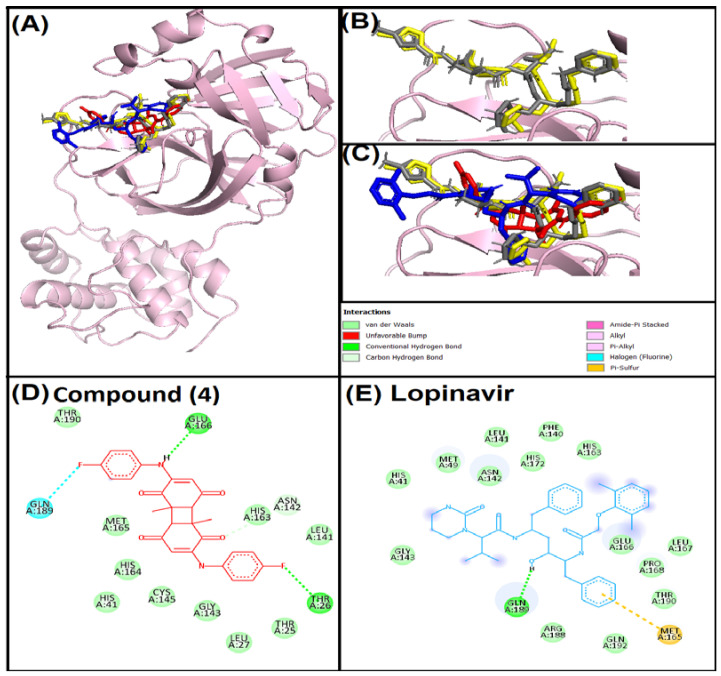
Superimposed image of docked ligands in the active site of 3CL^pro^ (PDB ID: 6LU7). (**A**) All the docked ligands (native ligand: Grey color; Redocked ligand: Yellow color; Compound (**4**): Red color; Lopinavir: Blue color) in the catalytic active site. (**B**) Superimposed zoom-in image of native ligand and redocked native ligand. (**C**) Zoom-in image of all the docked ligands. (**D**) Molecular interaction analysis of Compound (**4**) with amino acid residues. (**E**) Molecular interaction analysis of Lopinavir with amino acid residues.

Interaction between compound (**4**) and 3CL^pro^ revealed that compound (**4**) interacted with the active site of 3CL^pro^ (Figure 2). The binding mode of compound (**4**) was further compared with native ligand (N3) [36] and the protocol was standardized by re-docking the native ligand. Superimposition of the native ligand and re-docked native ligand at the active site confirmed the standardization of the protocol (Figure 2B). In addition, lopinavir was taken as a control and docked with the active site of 3CL^pro^. Compound (**4**), lopinavir and native ligand bound to the same position in the active site cavity of 3CL^pro^ (Figure 2C). Compound (**4**) showed van der Waals interactions, and hydrogen and halogen bondings with 3CL^pro^ (Figure 2D). Hydrogen bonding was observed between compound (**4**) and THR26, ASN142, GLU166 amino acid residues of 3CL^pro^, whereas GLN189 and THR26 were involved in halogen bonding. Ten amino acid residues of 3CL^pro^ showed van der Waals interactions with compound (**4**). On the other hand, lopinavir interacted through the hydrogen bond, van der Waals and pi-sulphur interactions with 3CL^pro^ (Figure 2E). GLN189 amino acid residue of 3CL^pro^ showed hydrogen bonding, while MET165 showed a pi-sulphur interaction. However, fourteen amino acids were involved in van der Waals interactions. GLU166, GLN189 and THR26 are important amino-acid residues for 3CL^pro^ active site targeting. Even re-docking with the native ligand also showed the involvement of GLU166 and GLN189 in hydrogen bonding. In common with present findings, some recent studies have also found the involvement of GLU166, GLN189 and THR26 in binding of active ligands with the active site of 3CL^pro^ [10,37,38].

**Figure 3:**
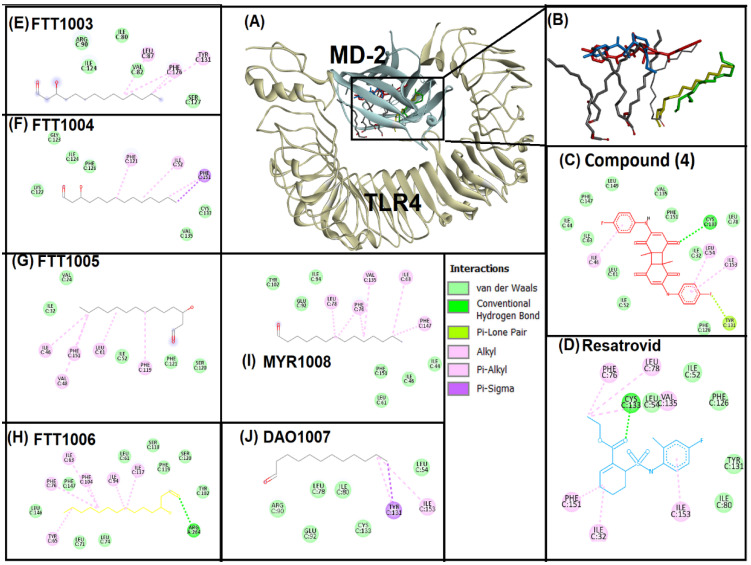
Superimposed image of docked ligands in the active site of TLR-4 (PDB ID: 3FXI). (**A**) All the docked ligands (Native ligand: Grey color; Redocked ligand: Yellow and Green color; Compound (**4**): Red color; Restrovid: Blue color) in the catalytic active site. (**B**) Zoom-in image of all the docked ligands. (**C**) Molecular interaction analysis of Compound (**4**) with amino acid residues. (**D**) Molecular interaction analysis of Resatrovid with amino acid residues. (**E**–**J**) Molecular interaction analysis of six lipid chains of lipopolysaccharides with amino acid residues.

The binding pocket of the TLR4-MD-2 complex was targeted for the present study. Myeloid differentiation factor 2 (MD-2) is a lipopolysaccharide sensing co-receptor for TLR4 [39]. TLR4 activation by spike protein of SARS-CoV-2 is also associated with MD-2 [40]. Thus, inhibition of the TLR4-MD-2 binding pocket is crucial to reduce inflammatory/neuro-inflammatory responses associated with COVID-19. In the present study, compound (**4**) and positive control (resatrovid) were docked to the binding pocket of TLR4-MD-2 (Figure 3). To standardize the protocol, native ligands, i.e., six lipid chains of lipopolysaccharides were re-docked with the binding pocket (Figure 3E–J). Superimposition of native ligands, compound (**4**) and restrovid revealed that all of them bind to the same vicinity of the binding pocket of TLR4-MD-2 (Figure 3A,B). Further interaction analysis was performed for compound (**4**) and restrovid (Figure 3C,D). Compound (**4**) interacted with the TLR4-MD-2 binding pocket via the hydrogen bond (CYS133), pi-lone pair (TYR131) and pi-alkyl (ILE46, LEU54, ILE153) interactions, whereas eleven amino acid residues were involved in van der Waals interactions with compound (**4**). In contrast, restrovid interacted with the binding pocket of TLR4-MD-2 through the hydrogen bond (CYS133), alkyl interactions (ILE32, LEU78, VAL135) and pi-alkyl ineractions (PHE76, PHE151, ILE153), while only five amino acids (ILE52, LEU54, ILE80, PHE126, TYR131) showed van der Waals interactions. Both compound (**4**) and restrovid showed strong hydrogen bonding with CYS133. Similarly, in other reports, mygalin, curcumin, nicotine and its metabolite cotinine also showed strong interaction with the CYS133 amino acid residue of the TLR4-MD-2 binding pocket [41,42].

**Figure 4:**
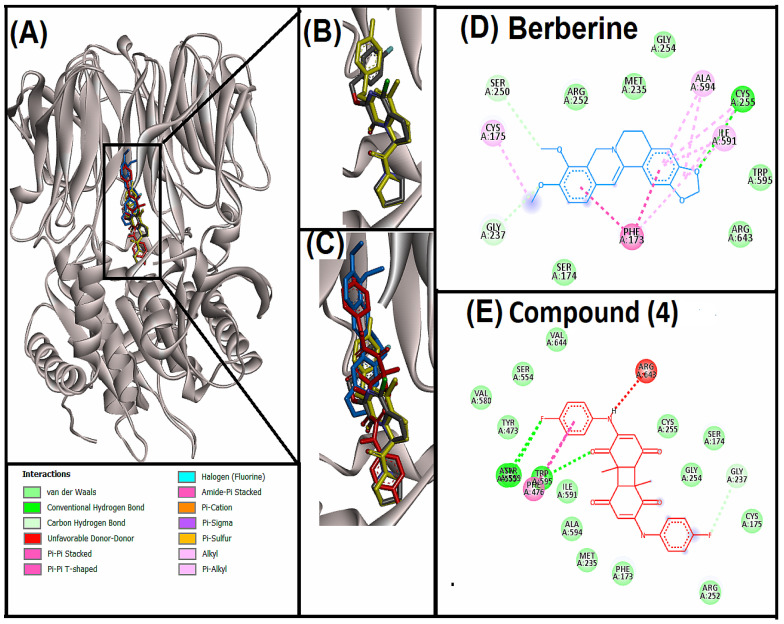
Superimposed image of docked ligands in the active site of PREP (PDB ID: 3DDU). (**A**) All the docked ligands (native ligand: Grey color; Redocked ligand: Yellow color; Compound (**4**): Red color; Berberine: Blue color) in the catalytic active site. (**B**) Superimposed zoom-in image of native ligand (GSK552) and redocked GSK552. (**C**) Zoom-in image of all the docked ligands. (**D**) Molecular interaction analysis of berberine with amino acid residues. (**E**) Molecular interaction analysis of compound (**4**) with amino acid residues.

PREP plays an important role in the neuro-inflammation and aggregation of α-synuclein [43,44]. In addition, it has been speculated that PREP also participates in COVID-19- associated neuro-inflammation. In fact, PREP acts as an important determinant of the angiotensin II level in tissues of SARS-CoV-2 patients [45]. Thus, in the present study our third target was PREP. Compound (**4**) and positive control (berberine) were docked to the active site of PREP (Figure 4). In addition, PREP was re-docked with the native ligand (GSK552) to validate the protocol (Figure 4B). Superimposition of compound (**4**), berberine, GSK552 and re-docked GSK552 indicated the positioning at the same site of the PREP (Figure 4C). Further, molecular interaction analysis for berberine and compound (**4**) was performed (Figure 4D,E). Compound (**4**) showed hydrogen and halogen bonding, pi-pi stacked and pi-pi T-shaped interactions and van der Waals interactions. ASN555, TRP595, TYR599 and GLY237 amino acids were involved in hydrogen and halogen bonding, whereas TRP595 and PHE476 were involved in pi-pi stacked and pi-pi T-shaped interactions. In comparison, berberine showed hydrogen bonding, pi-pi T-shaped, amide-pi stacked, alkyl, pi-alkyl interactions with PREP. CYS255, GLY237 and SER250 amino acids were involved in hydrogen bonding; PHE173 was involved in pi-pi T-shaped interaction; PHE173 and SER174 were involved in amide-pi stacked interaction; CYS255, ALA594, ILE591 and CYS175 were involved in alkyl interaction; and PHE173, CYS255, ILE591 and ALA594 were involved in pi-alkyl interactions. PREP catalytic triads consist of three amino acid residues, i.e., HIS680, ASP641 and SER554. However, the active site has different specificity pockets consisting of S1 (PHE476, ASN555, VAL580, TRP595, TYR599 and VAL644) which forms a convenient hydrophobic environment for aromatic rings of inhibitors, S2 (ARG643) which is not so specific, and S3 (PHE173, MET235, CYS255, ILE591 and ALA594) which consists of several nonpolar residues to form a strong hydrophobic region [46]. Importantly, compound (**4**) interacted with all the S3 amino acid residues and SER554 of the catalytic triad through van der Waals interactions, and most of the amino acid residues of S1 pocket i.e., ASN555, TRP595 and TYR599 via strong hydrogen bonding and PHE476 through pi-pi T-shaped interaction.

In addition, interaction of all the DTQ analogues with the target proteins is shown in Supplementary Table S1. Comparative interaction analysis of all the DTQ analogues with 3CL^pro^ showed that GLN110 amino acid residue was common in hydrogen bonding with compound (**1**), (**2**), (**5**), (**6**) and (**8**). However, SER158 was involved in the hydrogen bonding of compound (**3**) and (**7**). On the other hand, GLN189 formed a hydrogen bond with the control lopinavir. As hydrogen bonding is considered as an important parameter for strong interaction, compound (**4**) showed maximum hydrogen bonding (THR26, ASN142 and GLU166) compared to other analogues. As discussed above, CYS133 of TLR-4 played a crucial role in the interaction of compound (**4**) and resatrovid via the hydrogen bond. Whereas none of the other compound showed hydrogen bonding with TLR-4 except compound (**1**). Two amino acid residues, namely, ARG106 and SER184 of TLR-4 were involved in the hydrogen bonding of compound (**1**). PREP amino acid interaction analysis showed that all the DTQ analogues showed hydrogen bonding except compound (**3**) and (**7**); however, compound (**4**) showed strong hydrogen bonds with four amino acid residues of PREP.

### 3.5. Molecular Dynamic (MD) Simulation Analysis

Physicochemical properties, toxicity assessments and molecular docking analysis indicated that compound (**4**) was the best among the different analogues of DTQ. Thus, compound (**4**) was further subjected to dynamic simulation. 

A YASARA tool was used to execute MD simulation to gain insight into the ‘DTQ analogues-protein’ complex’s dynamism in solvent during a time-period of 100 ns. Simulation parameters were optimized and maintained throughout the simulation time, and simulations were triplicated for each target protein and complex. The eventual goal of the MD simulation analysis was to cognize the binding affinity and stability of the complex.

**Figure 5:**
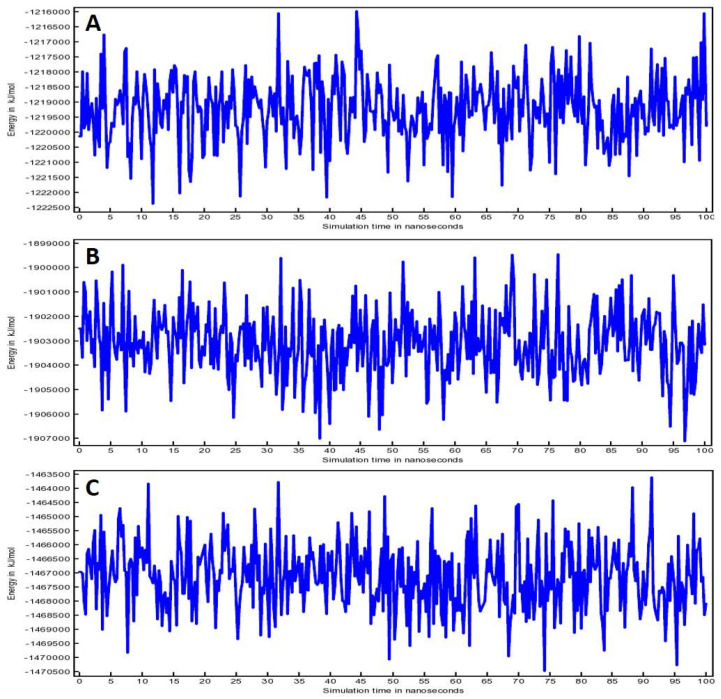
Total potential energy for (**A**) Compound (**4**)-3CL^pro^ complex, (**B**) Compound (**4**)-TLR4 complex and (**C**) Compound (**4**)-PREP complex.

To create potential energy (PE) plots, ‘AMBER14 force field’ was used (Figure 5). A sharp increase in energy is usually seen during the first few picoseconds of the simulation time when the simulation starts from frozen energy-minimization or ground-zero state. The ascent in energy might be due to part-stowing of kinetic energy as PE; however, PE is typically not diminished due to counter-ions during the long time-scale. They are situated predominantly with lower PE close to the charged solute groups, and after that they will disperse to upsurge the entropy and PE of the system. Figure 5A shows the plot of compound (**4**)-3CL^pro^ complex that oscillated between −1,216,000 and −1,222,500 kJ/mol. Whereas compound (**4**)-TLR4 complex (Figure 5B) showed the fluctuation from −1,899,000 to −1,907,000 kJ/mol. Compound (**4**)-PREP complex (Figure 5C) plot of PE indicated a variability from −1,463,500 to −1,470,500 kJ/mol. Notably, the variations were in an acceptable range for all the systems that predict the validity and stability of the simulation. 

**Figure 6:**
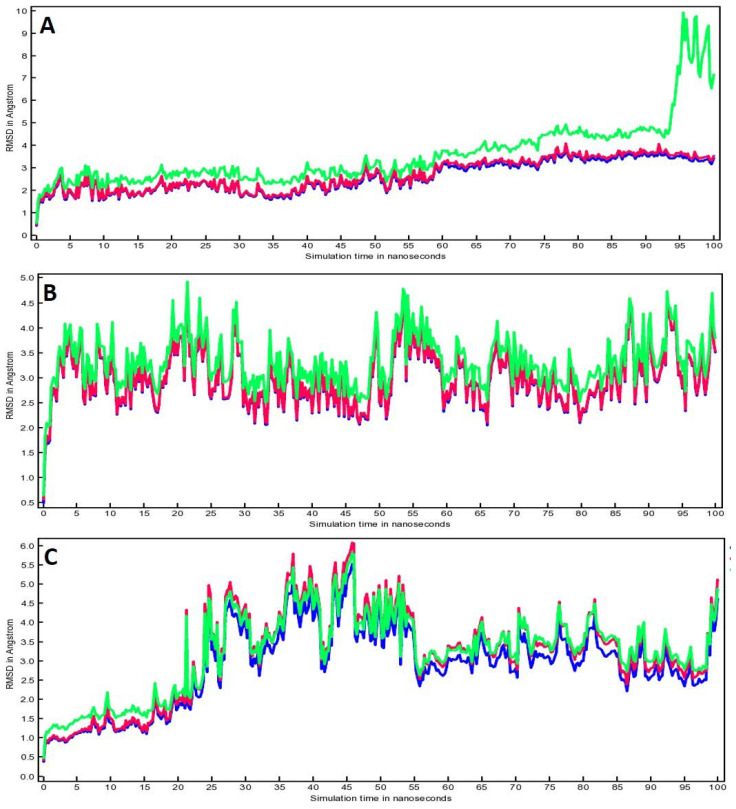
RMSD plots for (**A**) Compound (**4**)-3CL^pro^ complex, (**B**) Compound (**4**)-TLR4 complex and (**C**) Compound (**4**)-PREP complex. Here, green color line represents Cα RMSD, pink color line represents backbone RMSD and blue color line represents all heavy atoms RMSD.

Figure 6 shows the root mean square deviation (RMSD) for all the complexes that includes Cα RMSD (green color), backbone RMSD (pink color) and all heavy atoms RMSD (blue color). The RMSD plot for compound (**4**)-3CL^pro^ complex (Figure 6A) displayed that all RMSDs have minimal fluctuations until the end of 100 ns, except for Cα RMSD that showed fluctuations at the end of the simulation after 94 ns. Overall, the fluctuations for compound (**4**)-3CL^pro^ complex were in the range of 1 to 3 Å. Figure 6B showed that all RMSD for compound (**4**)-TLR4 complex were overlapping and showed fluctuations from 2.5 Å to 4.5 Å during the entire 100 ns run. Whereas the RMSD plot of compound (**4**)-PREP complex showed that all the RMSDs overlapped but started fluctuating from 2.5 Å to 5.5 Å during 23 ns to 55 ns. However, fluctuations were minimized after 55 ns until the end of the 100 ns run. In fact, RMSD measures the alteration in the protein backbone from its primary structural conformity to its final position with respect to time. Importantly, the average RMSD fluctuations in the present investigation did not exceed the acceptable range of 2 Å, and the results indicated that stable complexes were formed between the compound (**4**) and target proteins (3CL^pro^, TLR4, PREP).

**Figure 7:**
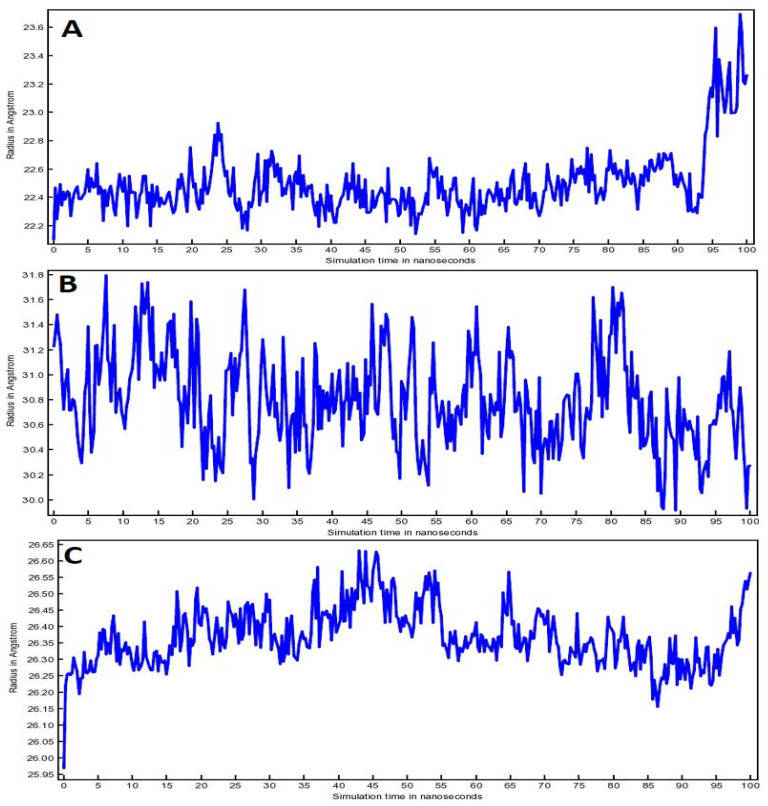
RoG plots for (**A**) Compound (**4**)-3CL^pro^ complex, (**B**) Compound (**4**)-TLR4 complex and (**C**) Compound (**4**)-PREP complex.

Figure 7 depicted the radius of gyration (RoG) of all the three complexes. The steadiness of RoG corresponds to the stability of the protein during the simulation run. The value of RoG depends on the protein atom’s distribution over its axis. In fact, the RoG estimation depends on the protein’s center of mass which represents the protein structure compactness throughout the simulation time. Compound (**4**)-3CL^pro^ complex showed little RoG fluctuations from 22.2 Å to 22.8 Å during 0 ns to 93 ns; however, fluctuation was increased to 23.6 Å after 93 ns (Figure 7A). RoG for compound (**4**)-TLR4 complex showed fluctuations from 30.0 Å to 31.8 Å during the entire 100 ns run (Figure 7B). Whereas minimal RoG fluctuations (26.25 Å to 26.65 Å) were observed in the case of compound (**4**)-PREP complex (Figure 7C). Overall, the fluctuations for compound (**4**) complexed with 3CL^pro^, TLR4 and PREP were within the acceptable range of 0.6Å, 1.8 Å and 0.4 Å, respectively.

**Figure 8:**
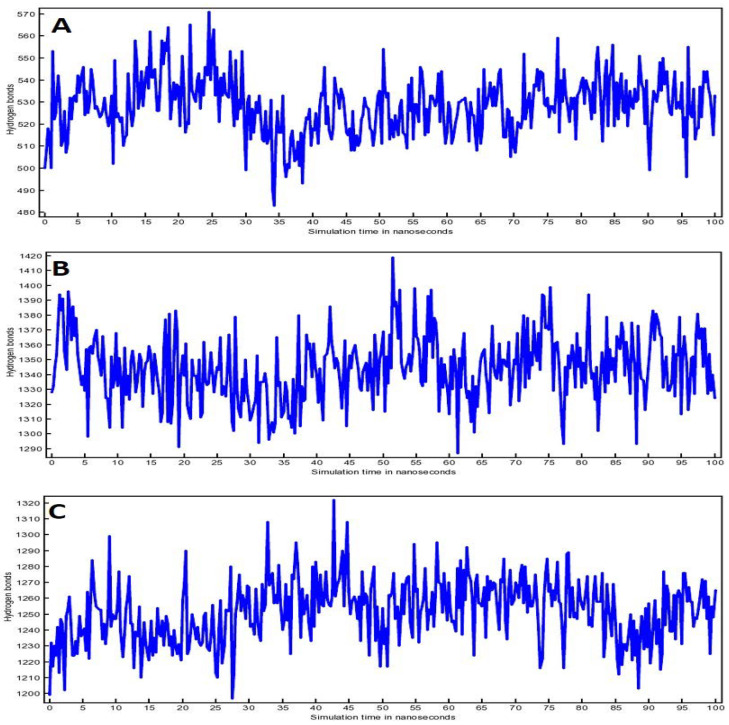
H bonds plot for (**A**) Compound (**4**)-3CL^pro^ complex, (**B**) Compound (**4**)-TLR4 complex and (**C**) Compound (**4**)-PREP complex.

In addition, effective protein folding and stability of target proteins were examined by the no. of H bonds during the 100 ns run. Figure 8 shows the increasing number of H bonds in correlation with the simulation time. It was evident from the plots that the no. of H bonds FLUCTUATED; however, the target proteins stability remains unaffected.

**Figure 9:**
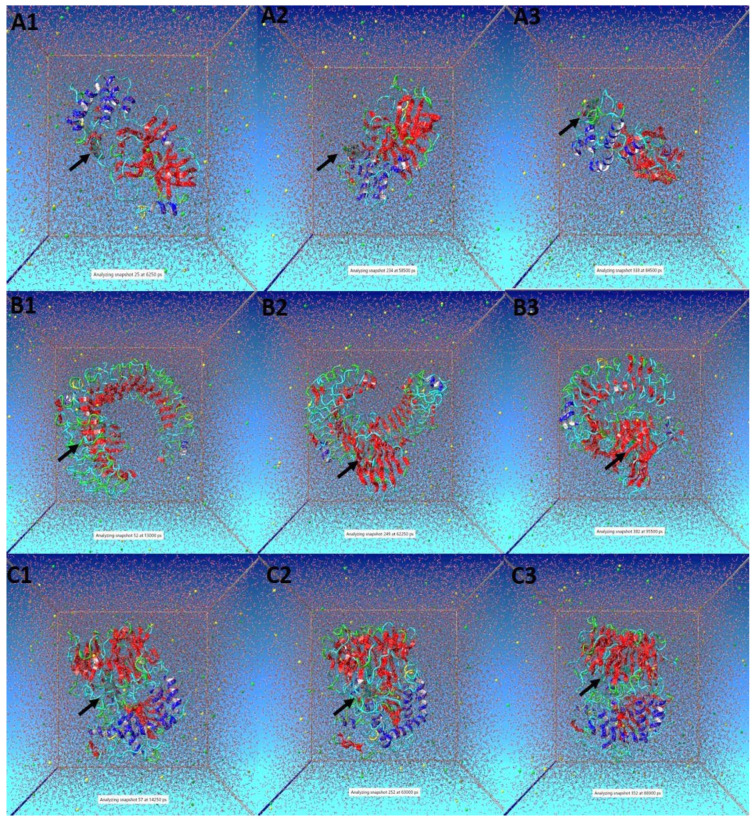
Snapshots of simulation runs for (**A**) Compound (**4**)-3CL^pro^ complex at different time intervals (A1, A2 and A3), (**B**) Compound (**4**)-TLR4 complex at different time intervals (B1, B2 and B3), and (**C**) Compound (**4**)-PREP complex at different time intervals (C1, C2 and C3). Here, black arrow indicates the location of Compound (**4**).

Few snapshots of the simulation at different time intervals were shown in Figure 9. Here, a black arrow was used to show compound (**4**) in complex with 3CL^pro^ (Figure 9A), TLR4 (Figure 9B) and PREP (Figure 9C), respectively. Overall, the findings suggested that compound (**4**) remained inside the binding cavity of 3CL^pro^, TLR4 and PREP in a stable conformation. It is worth mentioning that the authors earlier screened out DTQ from *Nigella sativa* as the multi-targeting compound against COVID-19 [10]. In addition, DTQ has shown the potential to inhibit the viral-host interaction for the SARS-CoV-2 infection [47]. Esharkawy et al. [48] checked the vitro anti-SARS-CoV-2 activity of DTQ and thymohydroquinone. They found that DTQ showed an IC_50_ value of 275.2 ng/mL against SARS-CoV-2-infected cells; however, it was cytotoxic against uninfected VERO-E6 cells. In the year 2021, DTQ was also reported to have antifungal, antioxidant, and anticancer potential [49]. Interestingly, the University of Kentucky Research Foundation has filed for a patent for anticancer activity against multi-drug-resistant human cancers of DTQ [50]. However, in the present study, DTQ analogues were designed by adding a certain functional group to prepare them as more potent and multitasking candidates against COVID-19 and its associated neurological complications. Among the different DTQ analogues, compound (**4**) showed the most promising results, that may be due to the addition of 4-fluoroaniline. In fact, adding 4-fluoroaniline can increase the hydrophobicity, bulkiness and anti-proliferative activity of the compound [18,51]. Yet to confirm the findings, in vitro experimental analysis is warranted. Nonetheless, the preliminary reports of the present study may help in providing a foundation for addressing issues pertinent to neuro-COVID.

## 4. Conclusions

Neuro-COVID has emerged as a problem in COVID patients since the start of the pandemic. The scientific community is working hard to address these issues; however, no major breakthrough has been achieved due to the complex nature of neurological complications associated with COVID. In the present investigation, DTQ analogues were designed and screened to find out the most promising candidate against SARS-CoV-2 and its associated neuro-targets using computational tools. The results suggested that DTQ analogue with incorporation of 4-fluoroaniline group [compound (**4**)] appears to be multi-potent against SARS-CoV-2 target (3CL^pro^), and neuro-targets (TLR4 and PREP). The physicochemical parameters, toxicity assessment and molecular docking approaches showed that compound (**4**) was not toxic, did not violate Lipinski’s rule of 5, showed better binding with all the target proteins in comparison to all other analogues and positive control compounds. Furthermore, compound (**4**) complex with all the proteins showed stability during the 100 ns trajectory of molecular dynamic simulation analysis. The predicted findings need confirmation via in vitro experimental studies. Nevertheless, the preliminary results of the present study may pave the way to develop more potent agents against neuro-COVID in the near future.

## Figures and Tables

**Table 1:** Prediction of physicochemical properties and toxicity potential.

DTQ Analogues /Control	Physicochemical Properties								Toxicity Potential			
	% Abs	TPSA	M.W.	cLogP	H-acc.	H-don.	R.B.	L.V.	Mut.	Tum.	Reprod.	Irrit.
Rule			<500	≤5	<10	<5	≤10	≤1				
Comp. (**1**)	85.43	68.3	244.2	0.5	4.0	0.0	0.0	0	None	None	None	None
Comp. (**2**)	85.43	68.3	486.2	3.8	4.0	0.0	2.0	0	None	None	None	None
Comp. (**3**)	85.43	68.3	397.3	3.6	4.0	0.0	2.0	0	High	None	None	None
Comp. (**4**)	77.15	92.3	462.5	3.0	6.0	2.0	4.0	0	None	None	None	None
Comp. (**5**)	83.19	74.8	330.4	−0.1	6.0	0.0	2.0	0	None	None	None	None
Comp. (**6**)	85.43	68.3	514.3	4.3	4.0	0.0	4.0	1	High	High	High	None
Comp. (**7**)	85.43	68.3	425.3	4.1	4.0	0.0	4.0	0	Low	High	High	None
Comp. (**8**)	85.43	68.3	328.4	2.7	4.0	0.0	2.0	0	None	None	None	None
Lopinavir	67.6	120.0	628.8	4.8	9.0	4.0	15.0	2	None	None	None	High
Resatrovid	81.12	80.8	361.8	2.8	5.0	1.0	5.0	0	High	None	High	None
Berberine	94.71	41.4	338.4	0.9	5.0	1.0	2.0	0	Low	Low	None	None

% Abs = % of oral absorption; TPSA = Total Polar Surface Area in Å^2^; M.W. = Molecular Weight in g/mol; cLogP = Logarithm of compound partition coefficient between n-octanol and water; H-acc. = Hydrogen bond acceptor; H-don. = Hydrogen bond donor; R.B. = Number of rotatable bonds; L.V. = Lipinki’s rule of five violation; Mut. = Mutagenic effect; Tum. = Tumorigenic effect; Reprod. = Reproductive effect; Irrit. = Irritant.

**Table 2:** Molecular docking results of DTQ analogues and control compounds interaction with different targets.

DTQ Analogues /Control	3CL^pro^	TLR-4	PREP
Comp. (**1**)	−7.2 kcal/mol	−7.3 kcal/mol	−7.3 kcal/mol
Comp. (**2**)	−7.6 kcal/mol	−8.4 kcal/mol	−7.6 kcal/mol
Comp. (**3**)	−7.7 kcal/mol	−8.4 kcal/mol	−7.6 kcal/mol
Comp. (**4**)	−8.5 kcal/mol	−10.8 kcal/mol	−9.5 kcal/mol
Comp. (**5**)	−6.3 kcal/mol	−7 kcal/mol	−7 kcal/mol
Comp. (**6**)	−7.4 kcal/mol	−8.2 kcal/mol	−7.2 kcal/mol
Comp. (**7**)	−7.4 kcal/mol	−8.1 kcal/mol	−7.4 kcal/mol
Comp. (**8**)	−7.4 kcal/mol	−8.3 kcal/mol	−7.9 kcal/mol
Lopinavir	−8.4 kcal/mol	-	-
Resatrovid	-	−7.3 kcal/mol	-
Berberine	-	-	−7.5 kcal/mol

## Data Availability

Not applicable.

## Supplementary Materials

The following supporting information can be downloaded at: https://www.mdpi.com/article/10.3390/life12071076/s1, Table S1: Interacting amino acids involved in the interaction of DTQ analogues with 3CLpro, TLR-4 and PREP. Amino acid(s) involved in hydrogen bonding are represented in bold font.
