# Supplementary material for: Dithymoquinone Analogues as Potential Candidate(s) for Neurological Manifestation Associated with COVID-19: A Therapeutic Strategy for Neuro-COVID
Source: Life (Basel). 2022 Jul 19;12(7):1076. doi: 10.3390/life12071076 (PMC9323060; doi:10.3390/life12071076)
Supplement: Supplementary file 1 [file life-12-01076-s001.zip › life-1766040-supplementary.pdf]

Supplementary Table S1: Interacting amino acids involved in the interaction of DTQ analogues with $3CL^{pro}$, TLR-4 and PREP. Amino acid(s) involved in hydrogen bonding are represented in bold font.

Target 1	Compound	Interacting Amino acids
$3CL^{pro}$	Comp. (1)	Phe8, Val104, Ile106, **Gln110,** Thr111, Asn151, Asp153, Ser158, Phe294
	Comp. (2)	Val104, Arg105, Ile106, Gln107, **Gln110,** Thr111, Asn151, Ser158, Phe294
	Comp. (3)	Phe8, Val104, Arg105, Ile106, Gln107, Gln110, Thr111, Asn151, Asp153, **Ser158,** Phe294
	Comp. (4)	Thr 25, **Thr 26,** Leu 27, His 41, Leu 141, **Asn 142,** Gly 143, Cys 145, His 163, His 164, Met 165, **Glu 166,** Gln 189, Thr 190
	Comp. (5)	Val104, Arg105, Ile106, Gln107, **Gln110, Thr111,** Asn151, Ser158, Phe294
	Comp. (6)	Val104, Arg105, Ile106, Gln107, **Gln110,** Thr111, Asn151, Asp153, Ser158, Phe294
	Comp. (7)	Phe8, Val104, Arg105, Ile106, Gln107, Gln110, Thr111, Asp153, **Ser158,** Phe294
	Comp. (8)	Val104, Arg105, Ile106, **Gln107, Gln110,** Thr111, Asn151, Ser158, Phe294
	Lopinavir	His 41, Met 49, Phe 140, Leu 141, Asn 142, Gly 143, His 163, Met 165, Glu 166, Leu 167, Pro 168, His 172, Arg 188, **Gln 189,** Thr 190, Gln 192
**Target 2**	**Compound**	**Interacting Amino acids**
TLR-4	Comp. (1)	Ser103, Phe104, **Arg106,** Ala107, Glu111, Thr112, Val113, Asn114, Thr115, His159, **Ser184,** Leu212, Glu266
	Comp. (2)	Val24, Ile32, Val48, Ile52, Phe76, Leu78, Glu92, Cys133, Val135, Phe151, Ile153
	Comp. (3)	Val24, Ile32, Val48, Ile52, Phe76, Leu78, Glu92, Cys133, Val135
	Comp. (4)	Ile 32, Ile 44, Ile 46, Ile 52, Leu 54, Leu 61, Ile 63, Leu 78, Phe 126, Tyr 131, **Cys 133,** Val 135, Phe 147, Leu 149, Phe 151, Ile 153
	Comp. (5)	Ile32, Leu54, Ile80, Phe126, Tyr131, Cys133, Phe151, Ile153
	Comp. (6)	Ile32, Ile52, Leu54, Leu61, Ile80, Cys133, Val135, Phe151, Ile153
	Comp. (7)	Ile32, Val48, Ile52, Phe76, Leu78, Glu92, Cys133, Val135
	Comp. (8)	Ile32, Ile52, Leu54, Leu78, Phe121, Cys133, Val135, Phe151, Ile153

	Resatrovid	Ile 32, Ile 52, Leu 54, Phe 76, Leu 78, Ile 80, Phe 126, Tyr 131, **Cys 133**, Val 135, Phe 151, Ile 153
**Target 3**	**Compound**	**Interacting Amino acids**
**PREP**	Comp. (1)	Glu169, Arg170, **Tyr187, Ser189,** Tyr211, **Gln219**
	Comp. (2)	Met67, Tyr71, Tyr484, Ser485, **Ser487, Arg488,** Gln577, Val689, Glu692, Val693
	Comp. (3)	Phe173, Ser174, Cys175, Asn188, Gln208, Met235, Gly237, Ala238, Ser250, Arg252
	Comp. (4)	Phe 173, Ser 174, Cys 175, Met 235, **Gly 237,** Arg 252, Gly 254, Cys 255, Tyr 473, Phe 476, Ser 554, **Asn 555,** Val 580, Ile 591, Ala 594, **Trp 595, Tyr 599,** Arg 643, Val 644
	Comp. (5)	Tyr484, Ser485, Ser487, **Arg488, Gln577,** His680, Lys684, Val689, Glu692, Val693
	Comp. (6)	Met67, Tyr71, Tyr471, Tyr484, Ser485, Val486, **Ser487, Arg488,** Gln577, Val689, Glu692, Val693
	Comp. (7)	Phe173, Cys175, Asn188, Met235, Gly237, Ala238, Ser250, Arg252
	Comp. (8)	**Pro34,** Asp35, Thr39, Lys40, Val43, Glu44, Asn47, Thr200, Asp639, Val674, Thr676
	Berberine	Phe 173, Ser 174, Cys 175, Met 235, **Gly 237, Ser 250,** Arg 252, **Cys 255,** Ile 591, Ala 594, Trp 595, Arg 643
